# Pesticide-induced multigenerational effects on amphibian reproduction and metabolism

**DOI:** 10.1016/j.scitotenv.2021.145771

**Published:** 2021-02-11

**Authors:** Oskar Karlsson, Sofie Svanholm, Andreas Eriksson, Joseph Chidiac, Johanna Eriksson, Fredrik Jernerén, Cecilia Berg

**Affiliations:** a Science for Life Laboratory, Department of Environmental Sciences, Stockholm University, Stockholm 114 18, Sweden; b Department of Environmental Toxicology, Evolutionary Biology Centre (EBC), Uppsala University, SE-752 36 Uppsala, Sweden; c Department of Pharmaceutical Biosciences, Uppsala University, Box 591, 75124 Uppsala, Sweden

**Keywords:** Transgenerational, Linuron, Anti-androgenic, Frog, *Xenopus tropicalis*, Paternal epigenetic inheritance, SCD-1

## Abstract

Underlying drivers of species extinctions need to be better understood for effective conservation of biodiversity. Nearly half of all amphibian species are at risk of extinction, and pollution may be a significant threat as seasonal high-level agrochemical use overlaps with critical windows of larval development. The potential of environmental chemicals to reduce the fitness of future generations may have profound ecological and evolutionary implications. This study characterized effects of male developmental exposure to environmentally relevant concentrations of the anti-androgenic pesticide linuron over two generations of offspring in *Xenopus tropicalis* frogs. The adult male offspring of pesticide-exposed fathers (F1) showed reduced body size, decreased fertility, and signs of endocrine system disruption. Impacts were further propagated to the grand-offspring (F2), providing evidence of transgenerational effects in amphibians. The adult F2 males demonstrated increased weight and fat body palmitoleic-to-palmitic acid ratio, and decreased plasma glucose levels. The study provides important cross-species evidence of paternal epigenetic inheritance and pollutant-induced transgenerational toxicity, supporting a causal and complex role of environmental contamination in the ongoing species extinctions, particularly of amphibians.

## Graphical abstract

**Graphical Abstract:**
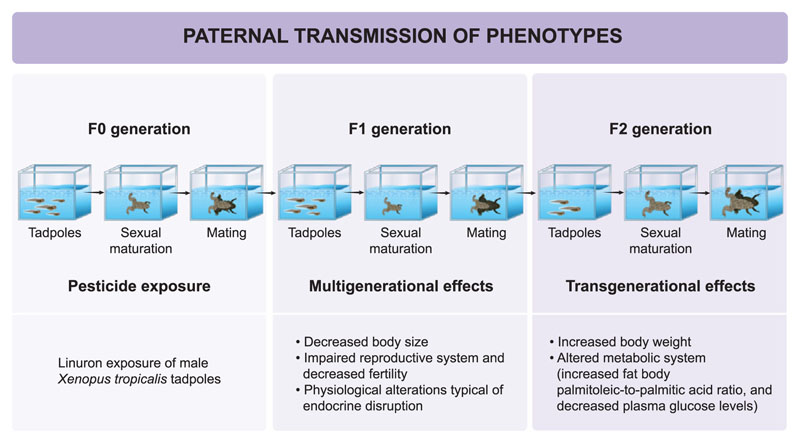

## 1 Introduction

Amphibians are suffering an unprecedented global decline, and almost half of all amphibian species are at risk of extinction. Suggested threats include habitat destruction, the introduction of foreign species, infectious diseases, climate change, and pollution (Falaschi et al., 2019; Gyllenhammar et al., 2009; Wu and Seebacher, 2020). Environmental contaminants linked to agricultural intensification, including endocrine-disrupting chemicals (EDCs), are reported to negatively affect gametogenesis and fertility after exposure during the larval stage (Gyllenhammar et al., 2009; Hayes et al., 2010). Several pesticides act as anti-androgens, and seasonal high-level agrochemical use overlaps with the period of amphibian breeding in the spring and thereby threatens the larvae during critical windows of development (Orton et al., 2011; Orton et al., 2018; Svanholm et al., 2020). We have recently demonstrated that early life exposure to the anti-androgenic pesticide linuron altered spermatogenesis and impaired fertility in male *Xenopus tropicalis* frogs (Orton et al., 2018). Exposures during sensitive early life stages may also increase the potential for multigenerational effects (Anway and Skinner, 2006; Perez and Lehner, 2019).

Growing evidence shows that not only genes but also the impacts of environmental influences may be passed down the generations through epigenetic mechanisms (DNA methylation, histone modifications, noncoding RNA) and alter an organism's phenotype without changing the DNA sequence (Heard and Martienssen, 2014; Perez and Lehner, 2019; Sharma et al., 2016). Interest in paternal transmission of life experience via epigenetic mechanisms partly originates from epidemiological studies associating paternal smoking with increased body mass index (BMI) in sons and relating a grandfather's food availability to mortality in grandsons (Pembrey et al., 2006; Vageroet al., 2018). Some studies suggest that early-life exposure to EDCs induces epigenetic alteration in mammals and impacts subsequent generations via epigenetic inheritance (Anway and Skinner, 2006). For example, *in utero* exposure to the anti-androgenic pesticide vinclozolin has been reported to impair spermatogenesis and male reproduction in third-generation offspring in rats via a possible epigenetic mechanism (Anway et al., 2005). The ecological, and possible evolutionary consequences, of man-made chemicals may therefore persist long after their release into the environment. However, unresolved issues within the field of epigenetic inheritance include: the prevalence of epigenetic trans-generational inheritance among organisms and cause-effect relationships between mechanism of action in exposed organisms and adverse effects in subsequent generations.

The aim of this study was therefore to investigate the effects of male developmental exposure to the pesticide, and anti-androgenic model compound, linuron over two generations of offspring in *Xenopus tropicalis* frogs at environmentally relevant concentrations. Linuron is used in many parts of the world, and measured environmental concentrations are around 10 μg/L (Woudneh et al., 2009), and in extreme cases up to 1.1 mg/L (Caux et al., 1998). Predicted environmental concentrations are in the range of 7–60 μg/L (EPA,2008). The most well-documented mechanism of toxicity for linuron is androgen receptor antagonism (Kojima et al., 2004; Orton et al., 2009; Wilson et al., 2009). In addition, linuron was recently shown to antagonize the thyroid receptor (Spirhanzlova et al., 2017). As both androgen and thyroid signaling are involved in testis development and regulation of lipid and carbohydrate metabolism, we analyzed endpoints related to male reproduction and metabolism.

## 2 Material and methods

### 2.1 Animals and housing

Adult Xenopus tropicalis frogs were obtained from Xenopus One (Dexter, MI, USA) and housed at the Evolutionary Biology Center, Uppsala University. Adult frogs were bred to obtain F0 tadpoles according to Berg, 2012. This is a follow-up study from Orton et al.,2018 in which the pesticide exposure of the F0 generation is described. In short, tadpoles were exposed via ambient water to 25.6 (±3.9) μg/L linuron (99.9% purity; CAS 330–55-2, Sigma-Aldrich, St. Louis, MO, USA) in 0.0008% acetone or to acetone only (0.0008%; hereafter referred to as control_F0_) using a semistatic system (50% water change three times per week) to maintain good water quality (Berg et al., 2009). Three replicate tanks were used for the control group and six tanks for linuron group. The treatment occurred from embryo Nieuwkoop and Faber (NF) stage 40 throughout the larval period until completion of metamorphosis (NF stage 66); thereafter, the F0 frogs were kept in clean water until they reached sexual maturity. The linuron concentration was analyzed by gas chromatography and mass spectroscopy throughout the exposure period (Orton et al., 2018). Water quality was assessed every other week for all tanks. Further details are provided in the Supplementary Information. The experiments were conducted according to protocols approved by the Uppsala Animal Ethical Committee and in accordance with the Swedish Legislation on Animal Experimentation (Animal Welfare Act SFS1998:56) and the European Union Directive on the Protection of Animals Used for Scientific Purposes (2010/63/EU).

### 2.2 Breeding of F1 and F2 male offspring

**Fig. 1:**
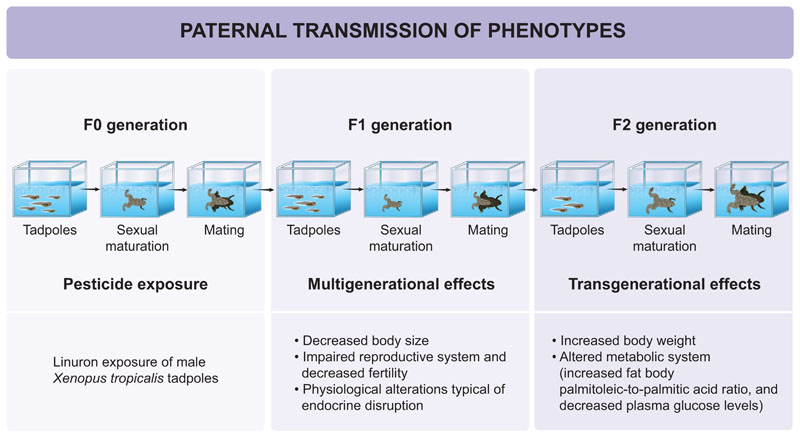
The overall study design and main findings.

The overall study design is presented in Fig. 1. The first-generation offspring (F1) were obtained from breeding 5 control_F0_ males and 11 linuron-exposed F0 males with naive females as described in Berg, 2012. In short, exposed males and naïve females were injected with human chorionic gonadotropin in the dorsal lymph sack 24 h before mating (200 IU) and right before mating (1000 IU). After the last injection the animals were placed in breeding tanks as described below to generate offspring. At 20 months of age, sexually mature F1 males (control_F1_ =12; linuron_F1_ = 32) were mated with naive females to generate the grand-offspring (F2) and enable studies of transgenerational effects (Heard and Martienssen, 2014; Perez and Lehner, 2019). When the adult F2 males reached 12 months of age, they (control_F2_ = 7; linuron_F2_ = 16) were mated with naive females to analyze fertility.

### 2.3 Analysis of sexual behavior and male fertility in the F1 and F2 generations

Male and female frogs were placed in pairs in breeding tanks and observed every 45th minute to determine the time to enter amplexus (mating position), time to spawning and total time in amplexus. The mating experiment was discontinued if the frogs did not enter amplexus within six hours. To study male fertility, a random subsample of the spawned eggs was collected using three glass Petri dishes on the bottom of the breeding tanks and transferred to holding tanks as described in Berg (2019). The fertilization rate was assessed by analyzing and comparing photographs taken immediately after egg collection and 26 h after the initiation of amplexus, when the hatched fertilized eggs could be distinguished by early embryo development (Orton et al., 2018). The numbers of laid eggs and developed larvae were calculated using ImageJ (NIH, Bethesda, MD, USA). The analyses were independently carried out by two persons who were unaware of to which experimental group each frog belonged.

### 2.4 Tissue collection, biometrics and secondary sexual characteristics

After breeding, the frogs were anaesthetized in tricaine (tricaine-methane sulfonate 0.3%, pH 7; Sigma-Aldrich, St. Louis, MO, USA), weighed, and photographed on top of a millimeter-scale paper (Nikon D70, objective AF micro Nikon 60 mm 1:2:8D), and after measurements of snout-vent length (SVL), fore limb width (secondary sex characteristic) and hind limb length (endpoint for disruption of the thyroid axis), killed by decapitation. The photographs of the nuptial pads were analyzed using ImageJ (NIH, Bethesda, MD, USA). The body mass index (BMI) was calculated using the body weight and SVL. The testes were collected and weighed to calculate the gonadosomatic index (GSI). Fat bodies (fat depots associated with the gonads) and liver samples were collected and stored at −80 °C until analysis. The left testis was fixed in neutral buffered formaldehyde (4% formaldehyde in phosphate buffer). The examiner was unaware of to which experimental group the frogs belonged.

### 2.5 Testis histology and counting of germ cells

The left testes were embedded in hydroxyethyl methacrylate for high-resolution morphological observation as previously described (Berg, 2012). Transverse, 2-μm-thick sections were cut at the anterior part of the testes using a microtome (Microm HM 360, Walldorf, Germany). The sections were stained with hematoxylin and eosin and evaluated at 40× magnification using a light microscope. On average, 10 and 16 seminiferous tubules per individual for the F1 and F2 generation, respectively, were randomly chosen for further histological evaluation using a grid overlay in ImageJ (NIH, Bethesda, MD, USA) (Gyllenhammar et al., 2009; Orton et al., 2018). For each tubule, the number of spermatogonia, number of spermatocytes in the largest germ cell nest, number of spermatozoa in the seminiferous lumen, total number of germ cell nests and germ cell nest stage were evaluated blindly to eliminate the risk of biased evaluation.

### 2.6 Plasma glucose analysis

After decapitation, trunk blood was collected at the decapitation site in heparinized tubes before centrifugation at 1000 ×g for 10 min. The plasma was collected and stored at −80 °C until analysis. Glucose levels were analyzed with enzymatic colorimetric methods using an Architect c4000 automated chemistry analyzer (Abbott Diagnostics, Lake Forest, IL,USA).

### 2.7 Fatty acid analysis of the liver and fat body

Lipids were extracted from 50 to 100 mg of liver and fat body tissue using a modified Folch procedure (Jernerén et al., 2015). An internal standard mix (heneicosanoic acid (21:0) and glyceryl triheptadecanoate (TG-17:0)) was added to all samples. A calibration curve was created using a series of fatty acid standards. The extracts and standards were subsequently transmethylated using methanolic-HCl (3 N) at 95 °C for 2 h, and the resulting fatty acid methyl esters were quantified by gas chromatography-mass spectrometry (Scion TQ, Bruker Daltonics Inc., Billerica, MA, USA) using a BPX70 column (25 m × 0.22 mm, 25 μm film, SGE, Weiterstadt, Germany) as previously described (Jerneren et al., 2015). Fatty acids were quantified by comparison with calibration curves, corrected for the presence of internal standards.

### 2.8 Statistical analysis

The results are presented as the mean ± SEM, and the data were analyzed using the nonparametric Mann-Whitney *U* test or Fisher's exact test for categorical variables. Differences were considered statistically significant at *p* values <0.05.

## 3 Results

### 3.1 F1 generation: body weight and characteristics

The adult male offspring of linuron-exposed fathers (linuron_F1_) demonstrated significantly altered body measures compared with the corresponding control_F1_ males (Table 1A). The linuron_F1_ males had a shorter SVL and lower body weight than the control_F1_ individuals. The body weight was also significantly lower in relation to the SVL, resulting in an 11% decrease in BMI compared with that in the control group. In addition, the weight of the fat body was decreased by 29% in male linuron_F1_ frogs. Linuron_F1_ frogs also had shorter hindlimbs and a reduced forelimb width compared to those in control_F1_ frogs (Table 1A).

### 3.2 F1 generation: metabolic analysis

The analyses of fatty acids and the desaturation index based on the 16:1/16:0 and 18:1/18:0 fatty acid ratios (Supplementary Tables S1–S2 and Fig. S1) in the fat body and liver revealed no significant difference between the groups. The analysis of plasma glucose showed no significant difference between the linuron_F1_ frogs (2.19 ± 0.12 mmol/L) and the control_F1_ frogs (1.90 ± 0.15 mmol/L).

### 3.3 F1 generation: reproductive system and fertility

The weight of the testes in the linuron_F1_ frogs was significantly higher relative to their body weight than that in the control_F1_ group, resulting in a 35% increase in the GSI. The area of the male breeding glands, the nuptial pads, was decreased by 23% compared to that in control_F1_ frogs (Table 1B). The breeding experiments revealed no significant differences between groups in terms of time to amplexus or the total time in amplexus. However, the linuron_F1_ males induced spawning significantly earlier than control_F1_ males (Table 1B). Notably, there was a significantly higher percentage of males with low fertility (< 15%) in the linuron_F1_ group (10/32) than in the control_F1_ group (0/12). The average fertility rates in the linuron_F1_ and control_F1_ groups were 30.8% and 44.9%, respectively (*p* = 0.0519).

### 3.4 F1 generation: analysis of spermatogenesis

**Fig. 2:**
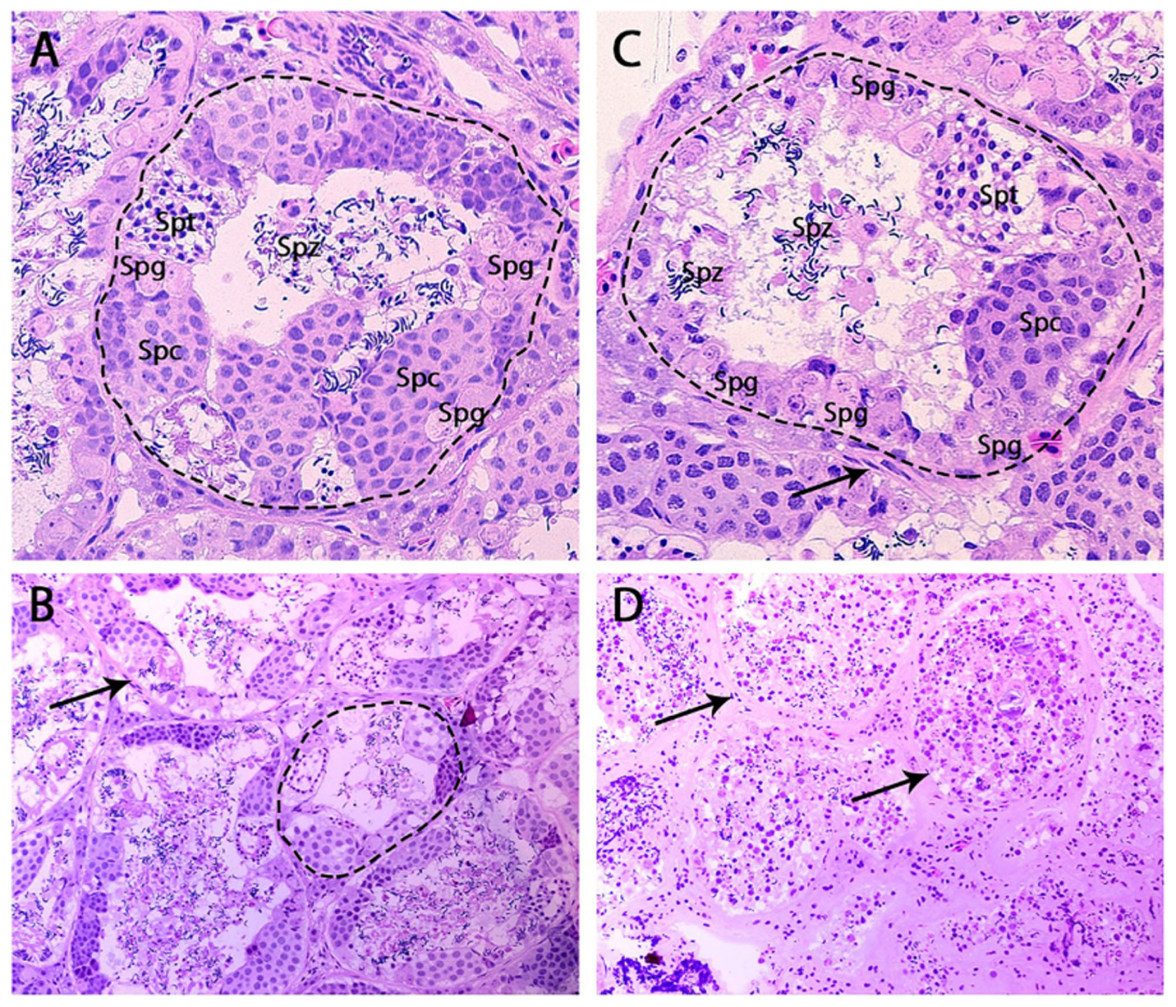
Histomicrographs showing seminiferous tubules (– – –) from 24-month-old *Xenopus tropicalis* A-B) F1 control male with all different germ cell stages and clearly structured, organized and distinguished seminiferous tubules with a thin layer of interstitial connective tissue and C) F1 male in the linuron group containing an elevated number of spermatogonia. D) F1 male in the linuron group with disorganized germinal epithelium in the seminiferous tubules, germ cells at different developmental stages scattered across the tubule, and thickened interstitial connective tissue. Arrows indicate interstitial connective tissue. Magnification 40× (A-B) and 20× (C-D). Spg = spermatogonia, Spc = spermatocytes, Spt = spermatids, Spz = spermatozoa.

The testis morphology was severely altered in eight out of 30 linuron_F1_ frogs. These testes lacked distinct germ cell nests, and both spermatocytes and spermatogonia were freely dispersed in the seminiferous tubule lumen. In addition, there was a higher abundance of interstitial connective tissue surrounding the seminiferous tubules (Fig. 2). These eight testes, as well as a control testis that lacked distinct germ cell nests, were excluded from further analysis. The detailed germ cell analysis showed a significant increase in the number of spermatogonia in the seminiferous tubules of linuron_F1_ frogs. They also had spermatocyte nests with an increased number of spermatocytes compared those in the control_F1_ group (Table 1C).

### 3.5 F2 generation: body weight and characteristics

Linuron_F2_ (the grand-offspring of linuron-exposed males) demonstrated an increased body weight and BMI (by 11%) compared with those in the control_F2_ group (Table 2A). The linuron_F2_ frogs also had longer hindlimbs than the controls (Table 2A). No significant group differences in the SVL, fat body weight, or forelimb measurements were detected.

### 3.6 F2 generation: metabolic analysis

There was a significant increase in the oleic acid (18:1) levels in the fat bodies, but not in the liver, of linuron_F2_ frogs (Supplementary Tables S3 and S4). The fat body palmitoleic acid-to-palmitic acid (16:1/16:0) ratio was higher than that in the control_F2_ group (Supplementary Fig. S2 A). A similar increase was demonstrated in the liver, but this increase was not statistically significant (Supplementary Fig. S2 C). The 18:1/18:0 fatty acid ratio was not altered in linuron_F2_ frogs (Supplementary Fig. S2 B and D). The plasma glucose levels were significantly (*p* = 0.0068) lower in the linuron_F2_ males (1.93 ± 0.14 mmol/L) than in the control_F2_ males (2.79 ± 0.22 mmol/L).

### 3.7 F2 generation: reproductive system and fertility

No effects on testes weight, GSI, or nuptial pad size were detected in the linuron_F2_ males compared with the controls_F2_. The breeding experiments revealed no significant differences in breeding behavior or fertility for linuron_F2_ males compared to those in the control_F2_ group (Table 2B). The average fertility rates were 35.0% and 35.1% in the linuron_F2_ and control_F2_ groups, respectively.

### 3.8 F2 generation: analysis of spermatogenesis

There was a significant decrease in the number of germ cell nests per seminiferous tubule in the linuron_F2_ males compared to the control_F2_ group (Table 2C). No other group differences were observed in the analysis of spermatogenesis in this generation.

## 4 Discussion

The diversity and quantity of man-made chemicals released into the environment are increasing at higher rates than other drivers of global environmental change (e.g. elevated atmospheric CO_2_), but relatively little attention has been given to assess how this affects biodiversity and ecosystem function (Bernhardt et al., 2017; Richmond et al., 2018; Wang et al., 2020). The present study provides evidence of transgenerational toxicity following environmentally relevant pesticide exposure in amphibians (Fig. 1).

The decreased fertility and perturbed spermatogenesis in the adult linuron_F1_ males are in line with the reported effects in their fathers (Orton et al., 2018). In addition, 27% of the linuron_F1_ males showed a severely abnormal testis histomorphology including pathological levels of interstitial connective tissue, a lack of distinct germ cell nests, and freely dispersed spermatocytes and spermatogonia in the seminiferous tubule lumen. Similar testis abnormalities have been demonstrated after developmental and adult exposure to anti-androgenic contaminants in *Xenopus laevis* frogs (Lee and Veeramachaneni, 2005; Rimayi et al.,2018), and in the F2 and F3 generations after in utero exposure in rats (Nilsson et al., 2018). The observed 35% increase in the GSI in linuron_F1_ males provides additional evidence of multigenerational reproductive system disruption. The reduced expression of androgen-dependent secondary sex characteristics (width of the forelimbs and the size of the nuptial pads) provides further evidence of disturbed androgen regulation in the linuron_F1_ males (Kelley and Pfaff, 1976; Orton et al., 2018). While the histological analysis revealed a significant decrease in the number of germ cell nests per seminiferous tubule in the linuron_F2_ males, no reduction in fertility, and no alteration to testes weight or the GSI, and the secondary sex characteristics similar to those in linuron_F1_ males was observed.

Alteration of the hindlimb length which was found in the F1 and F2 linuron males was also observed in the F0 males directly exposed to linuron during tadpole development (Orton et al., 2018) and implies transgenerational thyroid hormone disruption. Hindlimb development is highly dependent on thyroid hormones and used as an endpoint for disruption of the thyroid axis (OECD, 2009). Disrupted thyroid signaling may also be involved in the observed impact on body composition, as this hormonal system is important for the regulation of lipid and carbohydrate metabolism. The reduced body size of adult linuron_F1_ males compared to their controls is in line with a recent multigenerational study demonstrating that 12-month-old female exposure to triclosan or benzo(a)pyrene from the tadpole stage to adult age in *Xenopus tropicalis* resulted in a smaller F1 generation (Regnault et al., 2018). While these multigenerational effects do not exclude involvement of direct germ cell exposure in the progeny, transgenerational inheritance requires that the transmission cannot be ascribed to direct exposure to the environmental stimulus in the affected organism (Heard and Martienssen, 2014; Perez and Lehner, 2019). Transgenerational effects can be ascertained in the F2 generation in frogs, as they have externally developing embryos, whereas in mammals exposed *in utero*, effects in the F3 generation are required (Heard and Martienssen, 2014; Perez and Lehner, 2019).

Interestingly, the BMI significantly increased in adult linuron_F2_ males in comparison to the corresponding controls, and the plasma glucose concentration decreased, revealing a transgenerational impact of pesticide exposure on the metabolic system. As the linuron_F2_ males were heavier than the associated controls, without any difference in fat body weight or body length, it is likely that other major lipid deposits such as the cutaneous and subcutaneous adipose tissue have increased (Wygoda et al., 1987). In addition, the increased fat body ratio of palmitoleic acid to palmitic acid in the linuron_F2_ frogs indicates increased activity of stearoyl-CoA desaturase-1 (SCD-1), an enzyme that converts saturated fatty acids into monounsaturated fatty acids (Gong et al., 2011; Warensjo et al., 2006). In humans, a positive association between adipose palmitoleic acid concentrations and obesity has been found (Gong et al., 2011; Warensjo et al., 2006). Moreover, mice that lack SCD-1 are protected against diet-induced obesity, while increased SCD-1 activity is linked to obesity in animal models (Gong et al., 2011; Ntambi et al., 2002). Our results suggest similar correlations in frogs.

When comparing the observed effects between the studied generations, it is noticeable that the BMI and hindlimb length are reduced in linuron_F1_, while they increased in linuron_F2_ frogs. Similar changes in effect directions have been observed in previous studies, and may for example be linked to possible direct germ cell exposure in F1 frogs but not in F2 frogs, or to an adaption to the environment (Blanc et al., 2020; Perez and Lehner, 2019; Sales et al., 2017). However, more studies are warranted to study the underlying mechanisms for the opposite effect directions across generations.

Based on the known mechanistic targets of linuron, the reported effects in the F0 generation (Orton et al., 2018) and our present findings regarding down-stream effects and adverse outcomes in the F1 and F2 generations, we have outlined a plausible adverse outcome pathway (AOP; Supplementary Fig. S3). The perturbations of androgen and metabolism related endpoints observed in the linuron male F1 and F2 offspring and in F0 males directly exposed to linuron (Orton et al.,2018) are plausible down-stream effects of androgen receptor and thyroid receptor antagonism as molecular initiating events (MIEs; Supplementary Fig. S3). As both thyroid and androgen signaling are involved in testis development and regulation of lipid metabolism, it is possible that the observed adverse outcomes result from interaction between these two pathways. There are several challenges in developing AOPs over several generations, such as compensatory responses, interactions between multiple signaling pathways etc. However, the present study constitutes a first and important step to develop a multigenerational AOP for paternally transferred effects of anti-androgen/anti-thyroid chemicals in amphibians by characterizing key events and adverse outcomes over generations following a defined exposure with regards to substance, exposure level, mechanisms of action and window of exposure. As there is very little knowledge on multigenerational toxicity in amphibians, our work provides unique data on responses/potential biomarkers that enable inter-species comparisons to address their read-across potential in vertebrates in ecological risk assessment.

Given that anti-androgenic chemicals constitute a common type of EDCs (including pesticides) released into the environment (Orton et al., 2011) and that they can adversely impact the fitness of future unexposed generations, our findings may have profound ecological and evolutionary consequences. Our results support a causal and complex role of chemical exposure in the ongoing loss of vertebrate populations, particularly the rapid worldwide amphibian decline. Reduced fertility impacts on the population dynamics and other sub-lethal metabolic effects may possibly interact and aggravate the effects of other suggested drivers of vertebrate extinctions such as infectious disease and climate change (Karlsson et al., 2020; Pabijan et al., 2020). Our study further underscores the importance of considering multigenerational toxicity in chemical risk assessment, which is not the case in current chemical regulations. Transgenerational toxicity has now been demonstrated in mammals, birds, amphibians, fish, insects, and roundworms (Berg et al., 2011; Blanc et al., 2020; Guerrero-Bosagna et al., 2018; Sharma et al., 2016; Usal et al., 2021). To advance the development of appropriate risk assessment strategies it is crucial to better understand the consequences of multigenerational effects of chemicals and the impact of epigenetic inheritance. Both ecological studies in complex systems and well-controlled experiments to determine underlying mechanisms and modes of toxicity are critical to understand the role of man-made chemicals as drivers of biodiversity loss.

## Supplementary Material

**Appendix A. Supplementary data**

Supplementary data to this article can be found online at https://doi.org/10.1016/j.scitotenv.2021.145771.

Supplementary material

## Figures and Tables

**Table 1:** Biometrics and reproductive system variables including fertility in adult^a^ *Xenopus tropicalis* F1 males after paternal developmental exposure to linuron.

Variable	Control_F1_			Linuron_F1_		
	Mean	SEM	n	Mean	SEM	n
**A. Biometrics**						
SVL^b^ (mm)	45.69	0.64	7	43.47**	0.34	30
Body weight (g)	11.31	0.28	7	9.21***	0.22	30
BMI (kg/m^2^)	5.43	0.13	7	4.85***	0.05	30
Fat bodies (g)	1.00	0.05	7	0.71***	0.04	30
Hindlimb length (mm)	59.13	0.83	7	56.39*	0.57	30
Hindlimb length/SVL	1.29	0.01	7	1.30	0.01	30
Forelimb width (mm)	3.94	0.11	7	3.51**	0.06	30
Forelimb length (mm)	7.14	0.29	7	6.91	0.10	30

**B. Reproductive system**						
Testes weight (g)	0.030	0.003	7	0.032	0.001	30
GSI^c^	0.261	0.022	7	0.354**	0.013	30
Nuptial pad size (mm^2^)	18.41	0.87	7	14.18**	0.69	30
Time to amplexus (min)	120.0	11.5	12	94.2	8.9	32
Time to spawning (min)	217.5	9.3	12	167.3*	13.9	32
Time in amplexus (min)	236.3	27.8	12	237.7	21.6	32
Fertility rate (%)	44.9	4.7	12	30.8	4.3	32
Fertility rate < 15% (no.)	0 animals		12	10 animals*		32

**C. Germ cell analysis**						
Germ cell nests per seminiferous tubule (no.)	8.6	1.2	6^f^	8.6	1.8	22^f^
Spermatocyte nests (%)	68.3	3.2	6	71.7	1.3	22
Spermatid nests (%)	14.9	1.7	6	14.0	0.9	22
Spermatozoa nests (%)	16.8	2.8	6	14.3	1.0	22
Spermatogonia per seminiferous tubule (no.)	15.4	1.6	6	23.6*	1.9	22
Size of spermatocyte nests^d^	2.7	0.07	6	2.9**	0.03	22
Spermatozoa in lumen^e^	2.1	0.2	6	1.8	0.06	22

a Sexual behavior and fertility assessed at 20 months of age, biometrics, tissue collection and analysis at 24 months of age.

b Snout-vent length measured from the tip of the snout to the end of the cloaca.

c Gonadosomatic index, calculated as (weight of both testes/body weight) × 100.

d The size of the largest spermatocyte nest in each seminiferous tubule was determined using score numbers (1–3) based on the number of spermatocytes: 1 = 1–10, 2 = 10–20, 3≥20 spermatocytes/nest.

e The number of spermatozoa in the seminiferous tubular lumen was estimated using score numbers (0–3): 0 = no spermatozoa and 1 ≤50%, 2 = 50%, 3 ≥50% of the lumen was occupied by spermatozoa.

f Eight testes in linuronF1 and one control testis that lacked distinct germ cell nests, were excluded from further analysis.

* *p* < 0.05 compared to control animals (Mann-Whitney U test or Fisher’s exact test).

** *p* < 0.01 compared to control animals (Mann-Whitney U test or Fisher’s exact test).

*** *p* < 0.001 compared to control animals (Mann-Whitney U test or Fisher’s exact test).

**Table 2:** Biometrics and reproductive system variables including fertility in adult^a^ *Xenopus tropicalis* F2 males after grand-paternal developmental exposure to linuron.

Variable	Control_F2_				Linuron_F2_		
	Mean	SEM	n		Mean	SEM	n
**A. Biometrics**							
SVL^b^ (mm)	41.94	1.52	7		42.57	0.58	16
Body weight (g)	6.71	0.39	7		7.70*	0.22	16
BMI (kg/m^2^)	3.83	0.19	7		4.24*	0.05	16
Fat bodies (g)	0.43	0.05	7		0.44	0.03	16
Hindlimb length (mm)	51.87	2.66	7		58.65**	0.58	16
Hindlimb length/SVL	1.24	0.06	7		1.38*	0.013	16
Forelimb width (mm)	2.99	0.18	7		3.04	0.12	16
Forelimb length (mm)	7.44	0.41	7		7.81	0.12	16

**B. Reproductive system**							
Testes weight (g)	0.019	0.003	7		0.018	0.001	16
GSI^c^	0.282	0.03	7		0.236	0.01	16
Nuptial pad size (mm^2^)	6.96	0.66	7		7.25	0.46	16
Time to amplexus (min)	109.29	21.64	7		98.44	16.54	16
Time to spawning (min)	218.57	22.88	7		185.63	9.96	16
Time in amplexus (min)	199.29	61.21	7		272.81	31.14	16
Fertility rate (%)	35.1	14.6	7		35.08	5.9	16
Fertility rate < 15% (no.)	3 animals		7		4 animals		16

**C. Germ cell analysis**							
Germ cell nests per seminiferous tubule (no.)	7.9	0.2	7		6.4*	0.5	16
Spermatocyte nests (%)	69.1	3.0	7		68.4	1.6	16
Spermatid nests (%)	13.7	1.2	7		16.1	1.3	16
Spermatozoa nests (%)	17.2	2.1	7		15.5	1.2	16
Spermatogonia per seminiferous tubule (no.)	12.1	0.8	7		12.1	1.3	16
Size of spermatocyte nests^d^	2.7	0.04	7		2.7	0.02	16
Spermatozoa in lumen^e^	1.8	0.2	7		1.5	0.1	16

a All parameters assessed at 12 months of age.

b Snout-vent length. Measured from the tip of the snout to the end of the cloaca.

c Gonadosomatic index, calculated as (weight of both testis/body weight) *100.

d The size of the largest spermatocyte nest in each seminiferous tubule was determined by using score numbers (1–3) based on the number of spermatocytes: 1 = 1–10, 2 = 10–20, 3 ≥20 spermatocytes/nest.

e The number of spermatozoa in the seminiferous tubular lumen was estimated using score numbers (0–3): 0 = no spermatozoa and 1 ≤50%, 2 = 50%, 3 ≥50% of the lumen was occupied by spermatozoa.

* *p* < 0.05 compared to control animals (Mann-Whitney U test).

** *p* < 0.01 compared to control animals (Mann-Whitney U test).

## Highlights

- First evidence of paternal transgeneratio nal effects in amphibians (Xenopus t.)
- Induced by environmental concentrations of the anti-androgenic pesticide linuron
- Reproductive and endocrine impair ments in F1, metabolic disruption in F2 generation
