# Supplementary material for: Pesticide-induced multigenerational effects on amphibian reproduction and metabolism
Source: Sci Total Environ. Author manuscript; Available in PMC 2023 Sep 8. (PMC7615066; doi:10.1016/j.scitotenv.2021.145771)
Supplement: Supplementary material [file EMS133688-supplement-Supplementary_material.pdf]

SUPPLEMENTARY MATERIAL

Pesticide-induced multigenerational effects on amphibian reproduction and metabolism

Oskar Karlsson[1], Sofie Svanholm[2], Andreas Eriksson[2], Joseph Chidiac[3], Johanna Eriksson[3], Fredrik Jerneren[3], Cecilia Berg[2]

[1]Science for Life Laboratory, Department of Environmental Sciences, Stockholm University, Stockholm, 114 18, Sweden
[2]Department of Environmental Toxicology, Evolutionary Biology Centre (EBC), Uppsala University, SE-752 36 Uppsala, Sweden
[3]Department of Pharmaceutical Biosciences, Uppsala University, Box 591, 75124 Uppsala, Sweden

METHODS

Animals and housing

The tadpoles were fed Sera Micron (Sera, Heinsberg, Germany) for the first weeks. Sera Vipagran Baby (Sera, Heinsberg, Germany) and Tropical Fish food S (Aquatic Nature, Roeselare, Belgium) were added to the daily diet as the tadpoles developed. When the tadpoles completed metamorphosis, the diet was gradually altered to consist of Tropical Fish food S and Tropical Fish food M (Aquatic Nature, Roeselare, Belgium) and finally Tropical Fish food M only. Fully grown frogs were relocated to a recirculatory system and fed Royale Horizon 23 (Skretting, Wincham, UK). The water in the recirculatory system was cleaned through a carbon filter system and a biological filter, sterilized by UV light, and maintained at 25-26°C, pH 7.5-8.5, conductivity 500 µSv/cm and O_2 saturation >50%. The levels of ammonia, nitrite and nitrate were 0-0.5 mg/L.

The tadpoles generated from F0/F1 males were kept in 3 - 8 tanks (60 tadpoles in each) for the control and linuron lineage respectively. At metamorphosis, the F1 animals were re-arranged and moved to new tanks with increasing number of replicates as they grew (2-5 per lineage). They were moved to a re-circulatory tank system where they were kept until adulthood and subsequently separated according to sex. For the F2 animals, as they increased in size, a few months post metamorphosis, they were transferred to new tanks (6-14 per lineage) in a re-circulatory tank system where they were kept until adulthood and subsequently separated according to sex.

RESULTS

Table S1. Fat body fatty acid profiles in 24-month-old *Xenopus tropicalis* F1 males after paternal developmental exposure to linuron. Fatty acids were compared between control and linuron animals using Mann-Whitney U-test.

		Control			$Linuron_{F1}$		
**Fatty acid**		**Mean (%)**	**SEM**	**n**	**Mean (%)**	**SEM**	**n**
14:0	Myristic acid	10.59	1.40	7	11.42	0.44	30
16:0	Palmitic acid	21.64	1.42	7	22.25	0.54	30
16:1	Palmitoleic acid	4.031	0.52	7	3.44	0.12	30
18:0	Stearic acid	2.073	0.31	7	1.88	0.11	29
18:1	Oleic acid	29.24	1.72	7	29.16	0.76	30
18:2	Linoleic acid	15.00	0.62	7	15.93	0.31	30
18:3n-3	α-Linolenic acid	2.17	0.36	7	2.67	0.16	25
20:3n-6	Dihomo-γ-linolenic acid	0.87	0.34	3	0.67	0.05	22
20:4n-6	Arachidonic acid	0.86	0.24	4	0.62	0.05	15
20:5n-3	Eicosapentaenoic acid	1.98	0.23	7	19.04	0.09	30
22:5n-3	Docosapentaenoic acid	1.39	0.37	7	1.05	0.07	26
22:6n-3	Docosahexaenoic acid	4.43	0.58	7	3.56	0.19	29

Table S2. Liver fatty acid profiles in 24-month-old *Xenopus tropicalis* F1 males after paternal developmental exposure to linuron. Fatty acids were compared between control and linuron animals using Mann-Whitney U-test.

		Control			$Linuron_{F1}$		
**Fatty acid**		**Mean (%)**	**SEM**	**n**	**Mean (%)**	**SEM**	**n**
14:0	Myristic acid	3.29	0.32	7	3.36	0.13	30
16:0	Palmitic acid	40.47	3.80	7	40.38	1.26	30
16:1	Palmitoleic acid	3.98	0.77	7	2.99	0.21	30
18:0	Stearic acid	2.69	0.12	7	2.66	0.07	30
18:1	Oleic acid	21.63	2.79	7	21.88	1.13	30
18:2	Linoleic acid	11.43	1.12	7	12.13	0.40	30
18:3n-3	α-Linolenic acid	1.37	0.14	7	1.32	0.07	30
20:3n-6	Dihomo-γ-linolenic acid	0.89	0.14	7	0.98	0.04	29
20:4n-6	Arachidonic acid	2.02	0.30	7	2.05	0.10	29
20:5n-3	Eicosapentaenoic acid	3.65	0.27	7	3.54	0.10	30

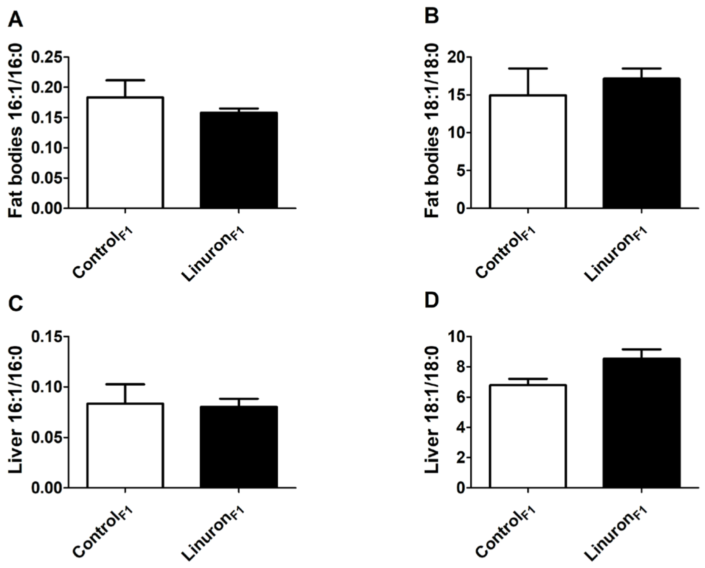

Figure S1. Desaturation index values based on fatty acids 16:1/16:0 (palmitoleic acid/palmitic acid) and 18:1/18:0 (oleic acid/stearic acid) in the fat body (A, B) and liver (C, D) of 24-month-old *Xenopus tropicalis* F1 males after paternal developmental exposure to linuron. Data are presented as the mean ± SEM. Control n=7; $linuron_{F1}$ n=28-30.

Table S3. Fat body fatty acid profiles in 12-month-old *Xenopus tropicalis* F2 males after grand-paternal developmental exposure to linuron. Fatty acids were compared between control and linuron animals using Mann-Whitney U-test. *p < 0.05 compared to control animals.

		**Control**			**Linuron$_{F2}$**		
**Fatty acid**		**Mean (%)**	**SEM**	**n**	**Mean (%)**	**SEM**	**n**
14:0	Myristic acid	5.52	0.28	7	5.21	0.19	16
16:0	Palmitic acid	20.50	1.95	7	19.69	0.72	16
16:1	Palmitoleic acid	5.76	0.42	7	7.04	0.48	16
18:0	Stearic acid	2.50	0.31	7	2.71	0.15	16
18:1	Oleic acid	24.04	1.19	7	28.06*	0.74	16
18:2	Linoleic acid	16.51	0.34	7	16.87	0.28	16
18:3n-3	α-Linolenic acid	3.04	0.27	7	3.21	0.11	16
20:3n-6	Dihomo-γ-linolenic acid	0.47	0.10	4	0.49	0.03	6
20:4n-6	Arachidonic acid	0.55	0.08	5	0.62	0.05	10
20:5n-3	Eicosapentaenoic acid	2.36	0.37	7	2.44	0.13	16
22:5n-3	Docosapentaenoic acid	1.29	0.10	5	1.16	0.09	14
22:6n-3	Docosahexaenoic acid	7.00	0.67	7	5.77	0.43	16

Table S4. Liver fatty acid profiles in 12-month-old *Xenopus tropicalis* F2 males after grand-paternal developmental exposure to linuron. Fatty acids were compared between control and linuron animals using Mann-Whitney U-test.

		**Control**			**Linuron$_{F2}$**		
**Fatty acid**		**Mean (%)**	**SEM**	**n**	**Mean (%)**	**SEM**	**n**
14:0	Myristic acid	2.54	0.19	7	2.36	0.24	16
16:0	Palmitic acid	43.51	2.56	7	42.91	1.77	16
16:1	Palmitoleic acid	8.05	1.19	7	11.68	1.81	16
18:0	Stearic acid	2.51	0.12	7	2.36	0.11	16
18:1	Oleic acid	17.94	1.58	7	17.36	1.54	16
18:2	Linoleic acid	7.38	0.66	7	6.33	0.33	16
18:3n-3	α-Linolenic acid	0.98	0.09	4	0.83	0.08	10
20:3n-6	Dihomo-γ-linolenic acid	1.04	0.11	3	0.92	0.13	10
20:4n-6	Arachidonic acid	1.76	0.30	7	1.47	0.14	15
20:5n-3	Eicosapentaenoic acid	1.41	0.14	6	1.29	0.13	16

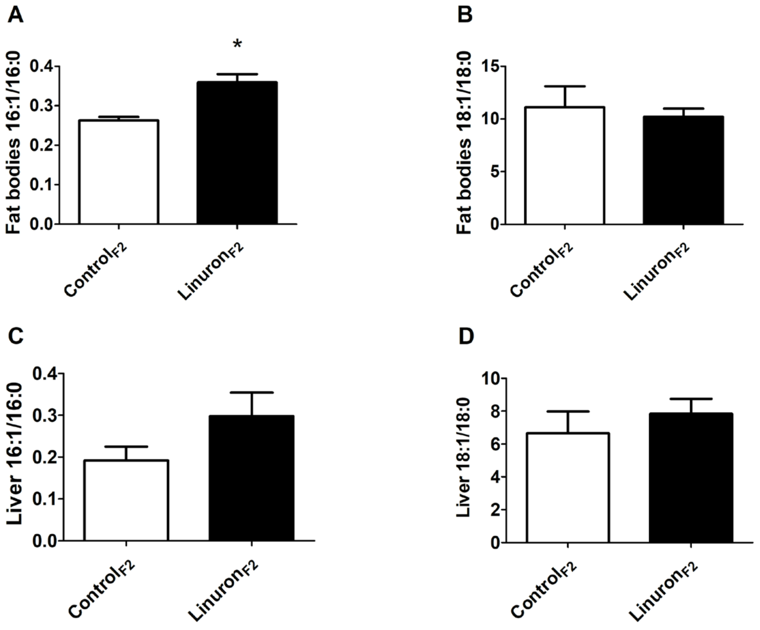

Figure S2. Desaturation index values based on fatty acids 16:1/16:0 (palmitoleic acid/palmitic acid) and 18:1/18:0 (oleic acid/stearic acid) in the fat body (A, B) and liver (C, D) of 12-month-old male *Xenopus tropicalis* F2 males after grand-paternal developmental exposure to linuron. *$p < 0.05$ compared to control animals (Mann-Whitney U-test), one outlier removed (Grubbs' test, $p < 0.05$, Z=2.02, considered outlier if >1.96 S.D. from mean). Data are presented as the mean ± SEM. Control n=6-7; linuron$_{F2}$ n=16.

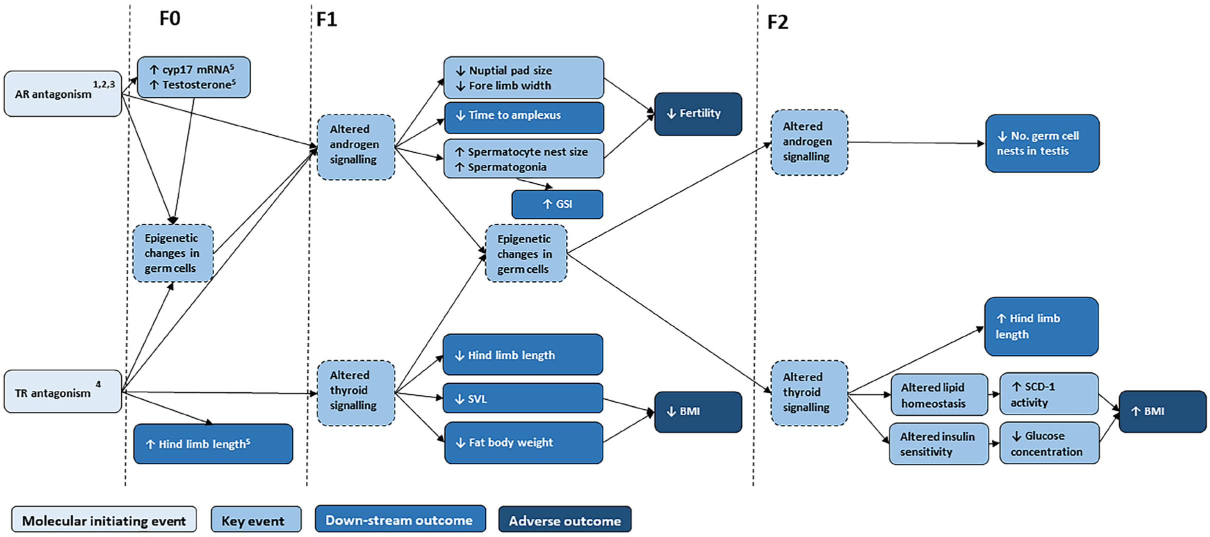

Figure S3. Suggested adverse outcome pathway (AOP) for multigenerational anti-androgenic and thyroid toxicity after paternal developmental exposure. Whole lines represent observed key events and dotted lines represent plausible key events. AR = Androgen receptor. TR = Thyroid receptor. Key event is here defined as either a molecular, cellular or tissue response as a consequent of the molecular initiating event leading to the adverse outcome. [1]Kojima et al., 2004, [2]Orton et al., 2009 ,[3]Wilson et al., 2009, [4]Spirhanzlova et al. 2017, [5]Orton et al., 2018
